# Identification of carboxymethyl (CM)-binding proteins derived from *Lolium multiflorum* pollen extract and antibody reactivity in Brazilian allergic patients

**DOI:** 10.1590/1414-431X2023e12957

**Published:** 2023-10-13

**Authors:** A.S. Correa, J.S. Miranda, L.A.R. Oliveira, P.F.S. Moreira, F.A.M. Vieira, J.P. Cunha-Junior, R.O. Resende, E.A. Taketomi

**Affiliations:** 1 Instituto de Ciências Biomédicas, Universidade Federal de Uberlândia, Uberlândia, MG, Brasil; 2 Departamento de Medicina, Universidade de Caxias do Sul, Caxias do Sul, RS, Brasil; 3 Instituto Oswaldo Cruz, Fiocruz, Rio de Janeiro, RJ, Brasil

**Keywords:** Allergen, Ion-exchange chromatography, Beta-expansin, Lolium multiflorum, IgE, IgG4

## Abstract

*Lolium multiflorum* grass is the major pollen allergen source in the southern region of Brazil, but most of its allergens remain poorly characterized. The aim of this study was to investigate antibody reactivity to *L. multiflorum* crude and carboxymethyl-ligand extracts in allergic patients and healthy individuals. Ion exchange carboxymethyl (CM) chromatography (CM-Sepharose) was used to isolate proteins (S2) from *L. multiflorum* crude extract (S1), which were assessed by SDS-PAGE. S1- and S2-specific IgE and IgG4 levels were measured by ELISA using sera from 55 atopic and 16 non-atopic subjects. Reactive polypeptide bands in S1 and S2 were detected by immunoblotting, and the most prominent bands in S2 were analyzed by mass spectrometry (MS-MS). Similar IgE and IgG4 levels were observed to both S1 (IgE median absorbance: 1.22; IgG4 median absorbance: 0.68) and S2 (IgE median absorbance: 1.26; IgG4 median absorbance: 0.85) in atopic subjects. S1 and S2 had positive correlations for IgE and IgG4 (IgE: r=0.9567; IgG4: r=0.9229; P<0.0001) levels. Homology between S1 and S2 was confirmed by IgE (84%) and IgG4 (83%) inhibition. Immunoblotting revealed that the 29-32 kDa band was recognized by 100% of atopic subjects in both S1 and S2. MS-MS analysis identified similarity profile to groups 1 and 5 grass allergens. This study revealed that carboxymethyl-ligand fraction played an important role for pollen allergy diagnosis by containing clinically relevant allergens and constituted a promising candidate for allergen-specific immunotherapy.

## Introduction

Seasonal allergic rhinitis, also known as hay fever, is an upper respiratory disease caused by the inhalation of airborne pollen, affecting allergic people particularly from spring to early summer, with well-defined symptoms, such as nasal itching, nasal congestion, sneezing, runny nose, and conjunctival hyperemia (1). In the past, it was thought that allergic rhinitis was an isolated and exclusive condition in airway allergy, but it has been currently shown that it is a systemic pathology with an intimate relationship with asthma and atopic dermatitis (2-5). Rhinitis affects approximately 50% of allergic patients around the world and both tree and grass pollens are among the most important sources of airborne allergens. Also, seasonal allergic rhinitis causes a great economic impact, in part because of the significant absenteeism rates directly associated with this disease (6-8).

*L. multiflorum*, Poaceae family, commonly known as Italian ryegrass, was introduced in Brazil by European immigrants, and it has been an important source for sensitizing individuals in the southern region of the country (9-11). Despite its importance, there is little evidence in the literature regarding the characterization of its major allergens, which is a reasonable concern for establishing diagnosis and treatment-specific strategies, particularly with immunodiagnostic tools and allergen-specific immunotherapy.

Pollen contains a wide range of proteins with distinct characteristics and functions, including β-expansins, which are also common in other grass pollen grains, as *Lolium perenne* and have been highlighted for more than half a century as the main components for inducing seasonal allergic rhinitis and seasonal asthma caused by grass pollen (12-14). Notably, β-expansins constitute up to 10% of the whole pollen grain, and Lol p 1, Poa p 1, Phl p 1 group 1 allergens derived from *Lolium perenne*, *Poa pratensis*, and *Phleum pratense*, respectively, are the most representative forms of this class of proteins in addition to group 5 allergens, which are exclusive to Pooideae subfamily. Many studies have demonstrated that these groups of allergens are immunodominant in grass pollen allergy by triggering robust IgE-mediated immune response. In addition, groups 2, 4, 7, 12, 13, 23, and 24 have also been described as mid-tier and minor allergens (15-18). Notably, Cyn d 1 was recently identified as 30 kDa-β-expansin major allergen from *Cynodon dactylon* (Bermuda grass) with high reactivity in patients with allergic rhinitis in subtropical regions, even though it has been responsible for the cross-reactivity with other grasses of the same region (19,20). In another study, groups 1 and 5 pollen isoforms were identified by 2D electrophoresis, and specific IgE antibodies found in sera demonstrated that they are essential for sensitization (7). Despite this, few studies have elucidated the role of pollen allergens as a potential application in the diagnosis and specific treatment of allergies. In this context, this study aimed to characterize protein fractions isolated from the total extract of *L. multiflorum* grass pollen, assessing IgE and IgG4 reactivity using sera of patients with seasonal allergic rhinitis.

## Material and Methods

### Subjects

A total of 71 subjects were recruited from the southern region of Brazil and distributed in two groups: atopic patients (AT) (n=55; 26 men/29 women; 29±10 years old) with a clinical history of pollinosis and positive skin prick test (SPT) to *L. multiflorum* total extract; and healthy non-atopic subjects (NAT) (n=16; 2 men/14 women; 21±12 years old), with no clinical history of pollinosis and negative SPT to *L. multiflorum* total extract (Table 1). The NAT group had a smaller sample size than the AT group because the immune response of those subjects showed a uniform negative SPT (no IgE reaction) to *L. multiflorum* extract. This study was approved by the Ethics Committee in Human Research of the Federal University of Uberlândia (protocol number 3.342.127), and subjects signed an informed consent form.

**Table 1:** Demographic and clinical characteristics of the subjects distributed according to clinical history of pollinosis and skin prick test.

Characteristics	Atopic n=55	Non-atopic n=16	P value
Gender^a^			<0.0001
Male	26 (47.3%)	2 (12.5%)	
Female	29 (52.7%)	14 (87.5%)	
Age^b^			0.1469
Median	29.5 (18-57)	21 (20-57)	
Mean wheal size (standard deviation, mm)^b^			<0.0001
*Lolium multiflorum*	10.9±3.465	0	
Skin prick test (%)^a^			<0.0001
Positive	55 (100%)	0 (0%)	
Negative	0 (0%)	16 (100%)	

^a^Chi-squared test or Fisher's exact test, when appropriate; ^b^Mann-Whitney test.

### Skin prick test

Immediate hypersensitivity response was assessed by SPT, as previously described (21). House dust mite (*Dermatophagoides pteronyssinus*, *Dermatophagoides farinae,* and *Blomia tropicalis*), dog (*Canis familiaris*), cat dander (*Felis domesticus*), fungus (*Alternaria alternata*), and grass pollen (*Cynodon dactylon*, *Phleum pratense*, *Paspalum notatum*, and *L. multiflorum*) allergen extracts were tested (FDA Allergenic, Brazil). Histamine hydrochloride 10 mg/mL and 0.9% sodium chloride were used as positive and negative controls, respectively. A wheal size 3 mm greater than the negative control was considered positive. Also, 10 mL of blood was collected from each subject and sera were obtained by centrifugation at 700 *g* for 10 min at room temperature.

### *L. multiflorum* extract

*L. multiflorum* was identified and registered by the University of Caxias do Sul Herbarium under specimen voucher HUCS 56105 available at https://specieslink.net/search/. Grass pollen was collected in Caxias do Sul, Rio Grande do Sul State, Brazil, during the flowering period of Italian ryegrass (September to November). Pollen content was sieved (0.50 mm pore/Tyler 32, Granutest-Telastem Sieves, Brazil) to eliminate potential contaminants. After separation, 5 g of grass pollen were mixed in 50 mL of 0.01 M phosphate-buffered saline (PBS), pH 7.2, containing 2 mmol/L phenylmethylsulfonyl fluoride (PMSF, Sigma-Aldrich, USA) and submitted to maceration at 4°C and extraction for 18 h at 4°C under orbital stirring. Subsequently, the solution was centrifuged at 15,000 *g* at 4°C for 30 min and the supernatant was concentrated (S1) and stored at -80°C. The protein content was determined by the Bradford method (22), using bovine serum albumin (BSA, Sigma) as standard.

### Ion-exchange chromatography

The *L. multiflorum* total extract S1 was separated by ion-exchange chromatography as previously described (13). Briefly, total extract S1 was thawed and centrifuged at 15,000 *g* at 4°C for 10 min. Then, the supernatant was dialyzed in equilibration buffer (20 mmol/L sodium citrate, pH 4.5) (Sigma) for 18 h at 4°C. Carboxymethyl (CM)-Sepharose resin (Cytiva Life Sciences, USA) was packed and equilibrated as recommended by the manufacturer, and extract S1 (2 mg/mL) was loaded onto the column. Unbound proteins were removed by washing with equilibration buffer and bound fractions were eluted with equilibration buffer supplemented with 200 mmol/L NaCl and UV absorbance was measured at 280 nm (BioTek Instruments, Inc., USA). The CM-binding fractions with the same electrophoretic profile were pooled (S2) and the protein content of S2 fraction was also determined as previously described.

### Measurement of IgE and IgG4 antibodies to *L. multiflorum* total extract (S1) and fraction (S2)

Serum IgE and IgG4 antibodies specific to *L. multiflorum* were measured by indirect ELISA as previously described (11). High-affinity microplates were coated with 50 μL of total extract S1 or fraction S2 at 20 μg/mL overnight at 4°C and blocked with PBS-Tween (T) containing 1% BSA for 1h at room temperature. Serum samples were diluted at 1:2 (IgE) or 1:5 (IgG4) and then incubated at 37°C for 2 h in a humid chamber. Subsequently, secondary human biotinylated anti-human IgE (1:500) or anti-human IgG4 (1:1000) (Sigma) antibodies were incubated for 1 h at 37°C, followed by streptavidin-peroxidase conjugate (Sigma) diluted at 1:500 for 30 min at room temperature. The reaction was developed by the addition of 0.01 M 2,2'-azino-bis-3-ethylbenzothiazoline-6-sulfonic acid (ABTS) (Life Technologies, USA) in 0.07 M citrate-phosphate buffer, pH 4.2, containing 0.03% hydrogen peroxide. Absorbance was monitored at 405 nm. The results are reported as absorbance. The cut-off was defined by the average absorbance value of the negative control sera plus 3 standard deviations.

### Inhibition ELISA

To evaluate the inhibition of S2 against extract S1, competitive inhibition ELISA was conducted as previously described (23). High-affinity microtiter plates were loaded with extract S1 (1 μg/well) in carbonate-bicarbonate buffer, and plates were then blocked with PBS-T containing 1% BSA for 1 h at room temperature. Subsequently, pooled serum from five positive samples determined by ELISA of the AT group were diluted (IgE: 1:2; IgG4: 1:5) and preabsorbed for 18 h at 4°C with 10-fold diluted S2 fraction. *Toxoplasma gondii* (Tg) extract was used as irrelevant antigen. Residual uninhibited antibody reactivity to extract S1 was measured by ELISA as described below and results are reported as the inhibition of S2 fraction in the absence of inhibitors (inhibition %).

### SDS-PAGE and immunoblotting

Total extract S1 and fraction S2 were submitted to sodium dodecyl sulfate-polyacrylamide gel electrophoresis (SDS-PAGE) prepared with 6% polyacrylamide for stacking and 14% for separation, under non-reducing conditions (24). The gels were stained with Coomassie blue and subsequently scanned (ChemiDoc XRS+, Bio-Rad, USA) and analyzed by Image Lab software (Bio-Rad Laboratories). Polypeptides were transferred to a nitrocellulose membrane (0.45 mm Millipore, USA) in buffer solution (25 mmol/L Tris, 192 mmol/L glycine, methanol), using a semi-dry transfer system (Multiphor II Electrophoresis Unit, Pharmacia LKB, Sweden) as described elsewhere (25). Nitrocellulose strips were blocked with PBS-T containing 2% skim milk (PBS-T-M) for 2 h at room temperature, followed by incubation overnight at 4°C under slow stirring with serum samples diluted 1:2 (IgE) or 1:5 (IgG4) in PBS. Then, the membranes were incubated with secondary human biotinylated anti-IgE (1:500) or anti-IgG4 (1:1000) antibodies diluted in PBS-T for 2 h at room temperature, followed by incubation with the streptavidin-peroxidase conjugate (Dako Corporation, USA) diluted 1:500 in PBS-T for 1 h at room temperature. After the final washing, 3,3-diaminobenzidine tetrahydrochloride (DAB) (Sigma-Aldrich) was added and bands were revealed.

### Mass spectrometry

Liquid chromatography tandem-mass spectrometry (LC-MS/MS) was carried out to identify polypeptide bands. Fraction S2 was subjected to SDS-PAGE as described above, and the bands of 12, 29-32, and 58-69 kDa were excised and stored in 10 mmol/L ammonium bicarbonate (AMBIC, Sigma). For sample preparation, the solution was discarded and a washing solution (50 mmol/L AMBIC supplemented with 40% acetonitrile - ACN, Sigma) was added; the sample was stirred on a Thermomixer (Eppendorf, USA) at 2,200 *g* for 30 min, the washing solution was discarded, and the steps were repeated 5 times. ACN was added to the gel, vortexed for 5 min, and discarded. Dithiothreitol 10 mmol/L (DTT, Bio-Rad) solution was added to the samples and mixed on a Thermomixer at 56°C at 2200 *g* for 45 min. Then, the DTT was discarded, followed by the addition of 55 mmol/L iodoacetamide solution (IAA, Thermo Fisher Scientific Inc., USA). After shaking for 30 min at room temperature in the dark, the IAA solution was discarded. Afterward, the samples were treated with AMBIC (50 mmol/L) and vortexed for 5 min, followed by disposal of the solution. Also, ACN was added to the sample, vortexed for 5 min, and then discarded. The samples were dried in a speed vacuum dryer, added to digestion buffer (100 ng of trypsin in 20 mL of 25 mmol/L AMBIC), and incubated for 16 h at 37°C at 800 *g*.

Peptide extraction was performed with the addition of trifluoroacetic acid (TFA, Thermo Fisher Scientific) to stop the digestion. The supernatant was collected in a LoBind microtube, mixed with an extraction solution (40% ACN in 0.1% TFA), and stirred for 15 min at room temperature. The process was repeated 4 times. The sample was dried in a speed vacuum dryer and resuspended in 0.1% TFA for desalting. Analyses were performed on LTQ-Orbitrap Velos ETD/Easy nanoLC II (Thermo Fisher Scientific). The peptides were separated on a C18RP column on a 95 min gradient. The instrumental conditions were checked using 100 fmol of a tryptic digest of BSA as standard. The carryover sample was completely removed between runs. The proteins were then filtered by ∼ 10 logp >15, selecting those with >3 unique peptides (UP) or at least 10% equality in the FASTA sequence, according to the UNIPROT database to select highly reliable non-redundant proteins. Alignment sequence analyses were performed using the Basic Local Alignment Search Tool (BLAST), followed by multiple sequence alignment analyses by CLUSTALW.

### Statistical analysis

Statistical analysis was performed using the GraphPad Prism software version 6.0 (GraphPad, USA). The frequencies of the antigenic bands recognized by serum antibodies in immunoblotting were subjected to comparative analyses between two proportions by the Z statistic. Kolmogorov-Smirnov normality test was used to determine distribution data model. The Mann-Whitney test was used to compare age, average wheal size, and the ELISA results. Pearson's test was used to determine correlation among data. All results were considered significant at a significance level of P<0.05.

## Results

### Protein profile of *L. multiflorum* total extract (S1) and fraction (S2)

**Figure 1:**
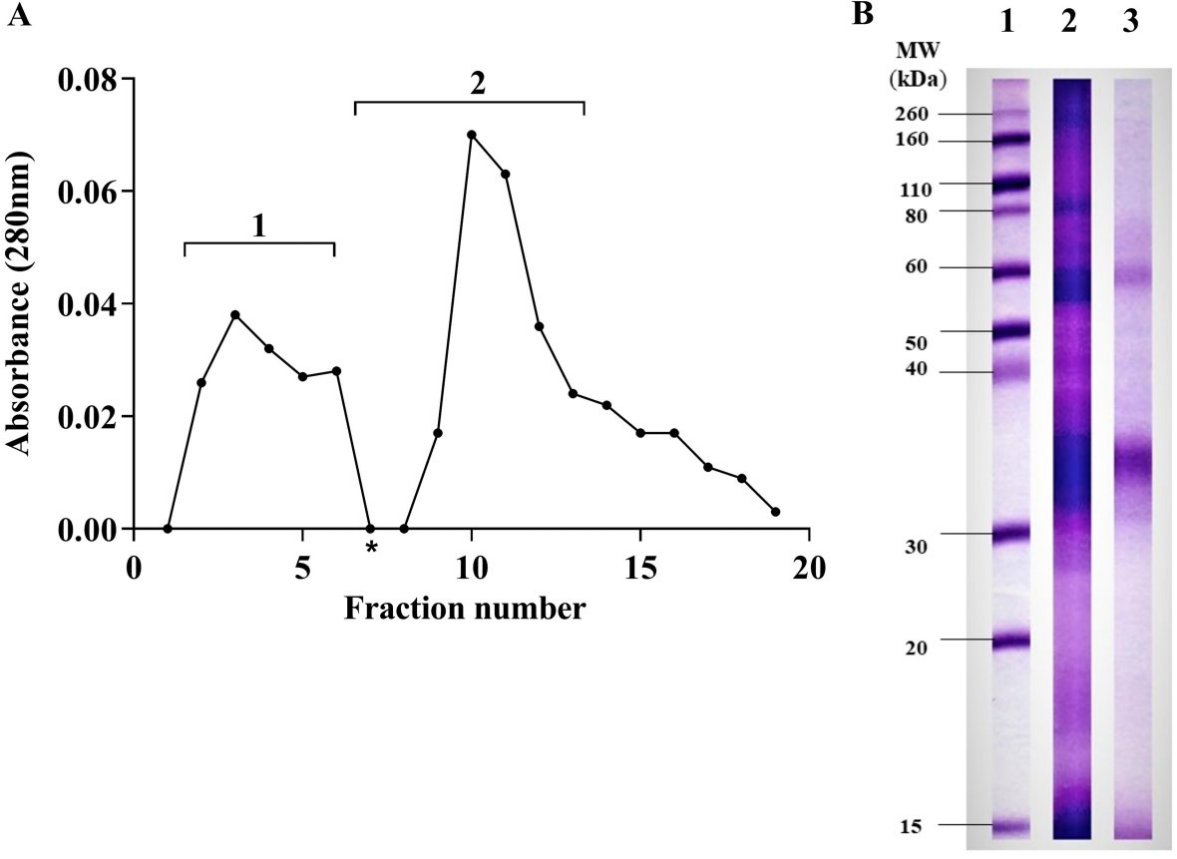
**A**, Fractionation profile of *Lolium multiflorum* total extract by ion-exchange carboxymethyl (CM) chromatography. Void volume (1) and fraction S2 (2) eluted with 20 mM NaCl. *Start of the elution. **B**, SDS-PAGE stained with Coomassie brilliant blue. Standard molecular weight values (lane 1), total extract S1 (lane 2), and fraction S2 (lane 3).

Protein content of all samples was determined by the Bradford method using BSA as a standard curve with correlation coefficient (r^2^) of 0.996, using cubic curve fit calculated by applying the following equation: Absorbance = 1.575 + 2.499(Conc) - 0.003899[(Conc)^2^] + 2.025 ×10^-6^[(Conc)^2^] + [(Conc)^3^]. Figure 1A shows the fractionation profile of *L. multiflorum* extract by ion exchange-chromatography. Peak 1 indicates proteins that were not bound to CM-sepharose resin and peak 2 represents resin-binding fractions that were eluted with 200 mmol/L NaCl.

The electrophoretic profiles of the total extract S1 and fraction S2 are shown in Figure 1B. The major components that were identified in the total extract S1 (protein content, 2,000 μg/mL) were the 12, 29-32, and 58 kDa bands. In addition, bands ranging from 17 to 145 kDa were also seen in the extract S1. The analysis of the fraction S2 revealed predominantly Coomassie-stained bands of 32 and 58 kDa and minor bands ranging from 12 to 69 kDa. The fraction S2 (protein content, 500 μg/mL) was particularly enriched with 29-32 kDa components.

### Specific IgE and IgG4 antibodies against total extract S1 and fraction S2 from *L. multiflorum*

**Figure 2:**
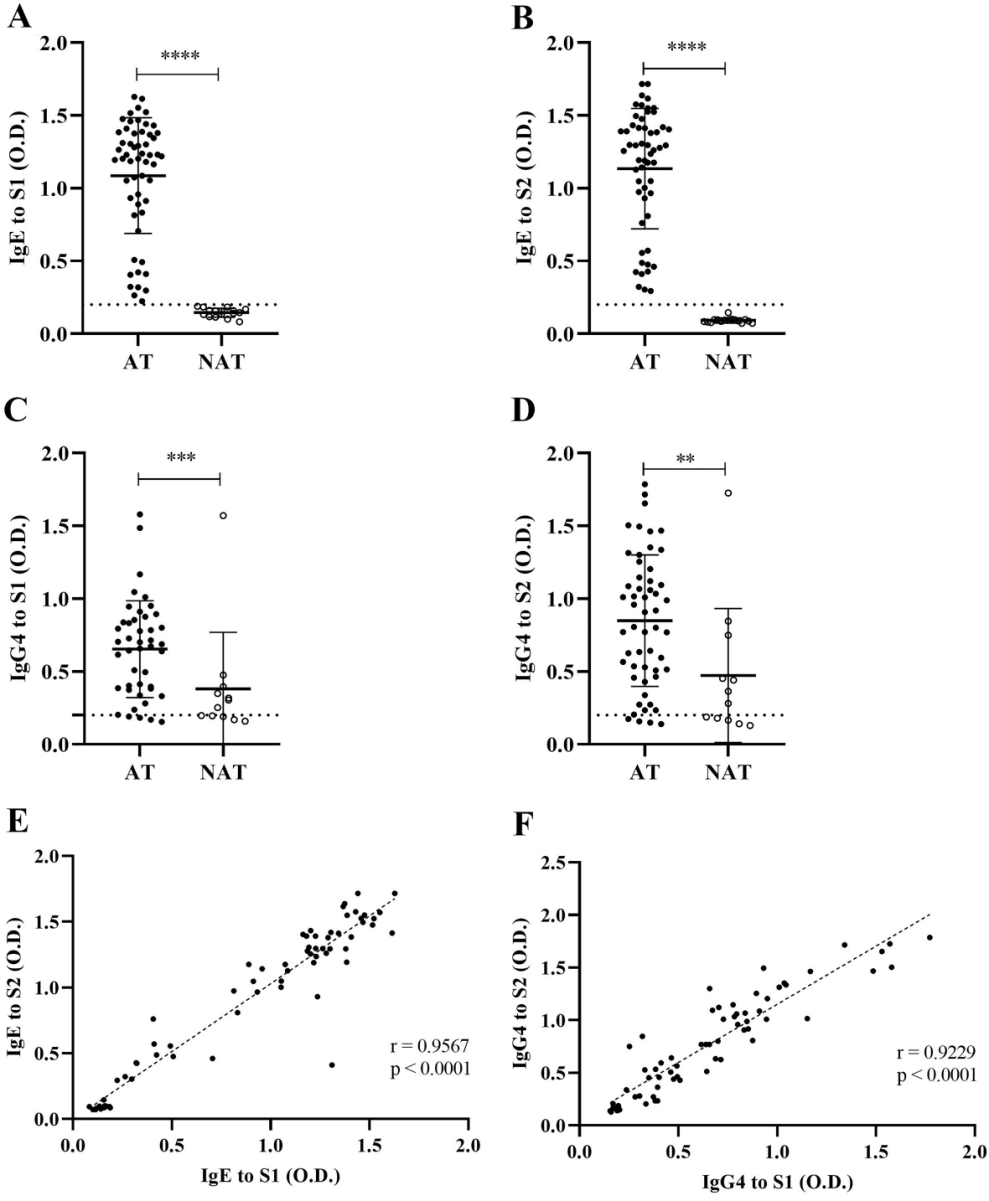
Levels of IgE and IgG4 specific to the *Lolium multiflorum* total extract (S1) (**A** and **C**) and carboxymethyl (CM)-binding fraction (S2) (**B** and **D**) in sera from 55 atopic patients (AT) with positive skin prick test to *L. multiflorum* and 16 non-atopic subjects (NAT), determined by ELISA. Differences between groups were compared by the Mann-Whitney test. Correlation coefficients (r) between levels of IgE (**E**) and IgG4 (**F**) specific to the total extract S1 and fraction S2 were calculated by Pearson's correlation coefficient test. Data are reported as absorbance (optical density (O.D.)). Median and positive threshold (0.2) are indicated by horizontal bars and dashed lines, respectively. **P<0.01, ***P<0.001, ****P<0.0001.

The levels of serum IgE against *L. multiflorum* total extract (S1) were significantly higher in AT patients (median=1.22; 100%) compared to NAT subjects (median=0.15; 0%) (P<0.0001) as shown in Figure 2A. As expected, NAT subjects presented undetectable levels of IgE antibodies anti-S1. Likewise, levels of serum IgE against fraction S2 were significantly higher in AT patients (median=1.26; 96.4%) than in NAT subjects (median=0.09; 0%) (P<0.0001), as shown in Figure 2B. In addition, as shown in Figure 2E, a strong correlation (r=0.9567; P<0.0001) was found when comparing serum IgE antibody reactivity to extract S1 and fraction S2 from all AT and NAT individuals.

The levels of serum IgG4 against *L. multiflorum* total extract (S1) were significantly higher in AT patients (median=0.68; 100%) compared to NAT subjects (median=0.27; 92.7%) (P=0.0019), as shown in Figure 2C. Also, levels of serum IgG4 against fraction S2 were significantly higher in AT patients (median=0.85; 96.7%) than in NAT subjects (median=0.32; 0%) (P=0.0041), as shown in Figure 2D. Additionally, a strong correlation (r=0.9229; P<0.0001) was found when comparing serum IgG4 antibody reactivity to the total extract S1 and fraction S2 from all AT and NAT individuals, as evidenced in Figure 2F.

### Extract S1-specific IgE and IgG4 antibodies inhibited by fraction S2

**Figure 3:**
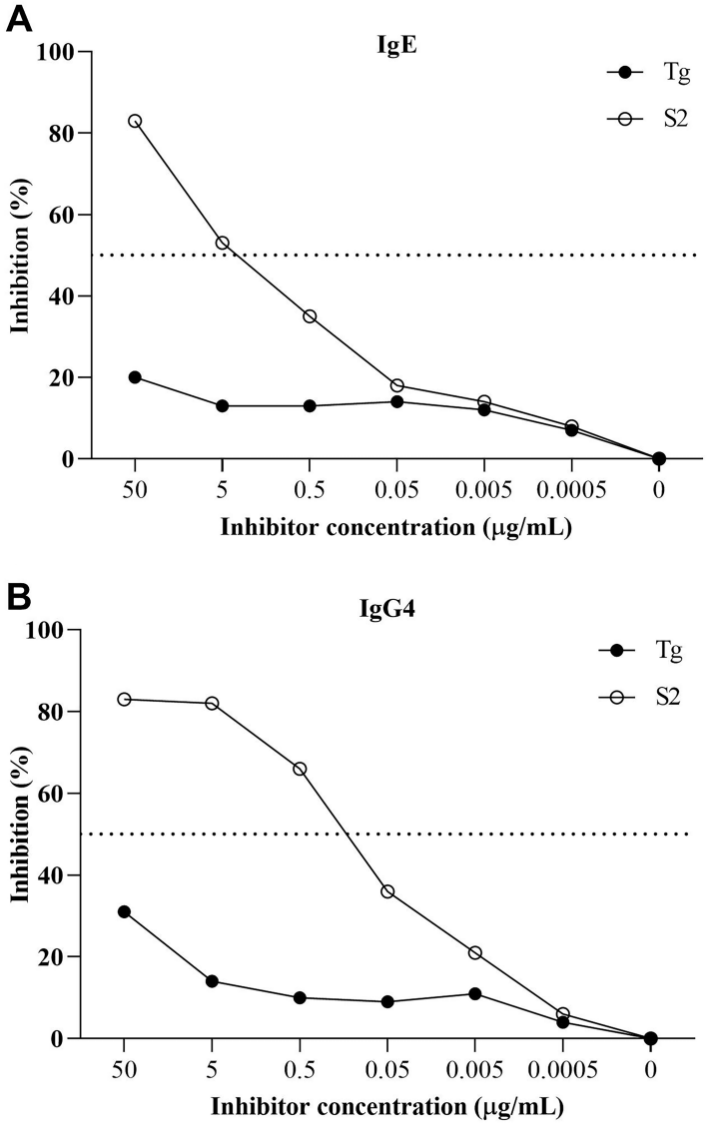
IgE (**A**) and IgG4 (**B**) inhibition curves using *Lolium multiflorum* total extract (S1) on the solid phase, by ELISA. Pooled sera from atopic patients were preabsorbed with different concentrations of CM-binding fraction derived from *Lolium multiflorum* (S2) or *Toxoplasma gondii* (Tg) as an irrelevant antigen (control). Dashed lines represent the half-maximal inhibitory concentration (IC50).

IgE and IgG4 cross-reactivity to S1 extract was assessed by inhibition ELISA using S2 fraction. Tg was used as an irrelevant antigen. High homology was observed between extract S1 and fraction S2 for IgE (84%) of pooled sera from the AT group (Figure 3A) and for IgG4 (83%) (Figure 3B) at the maximum concentration of 50 μg/mL of inhibitor. According to Figure 3A, 4.7 µg of the inhibitor was needed to inhibit 50% of IgE binding, while 0.37 µg of the inhibitor was necessary to inhibit IgG4 binding, as shown in Figure 3B. Tg exhibited low reactivity for IgE (20%) and IgG4 (31%) at the maximum concentration used.

### Allergens in *L. multiflorum* total extract S1 and fraction S2 recognized in IgE and IgG4 immunoblotting

**Figure 4:**
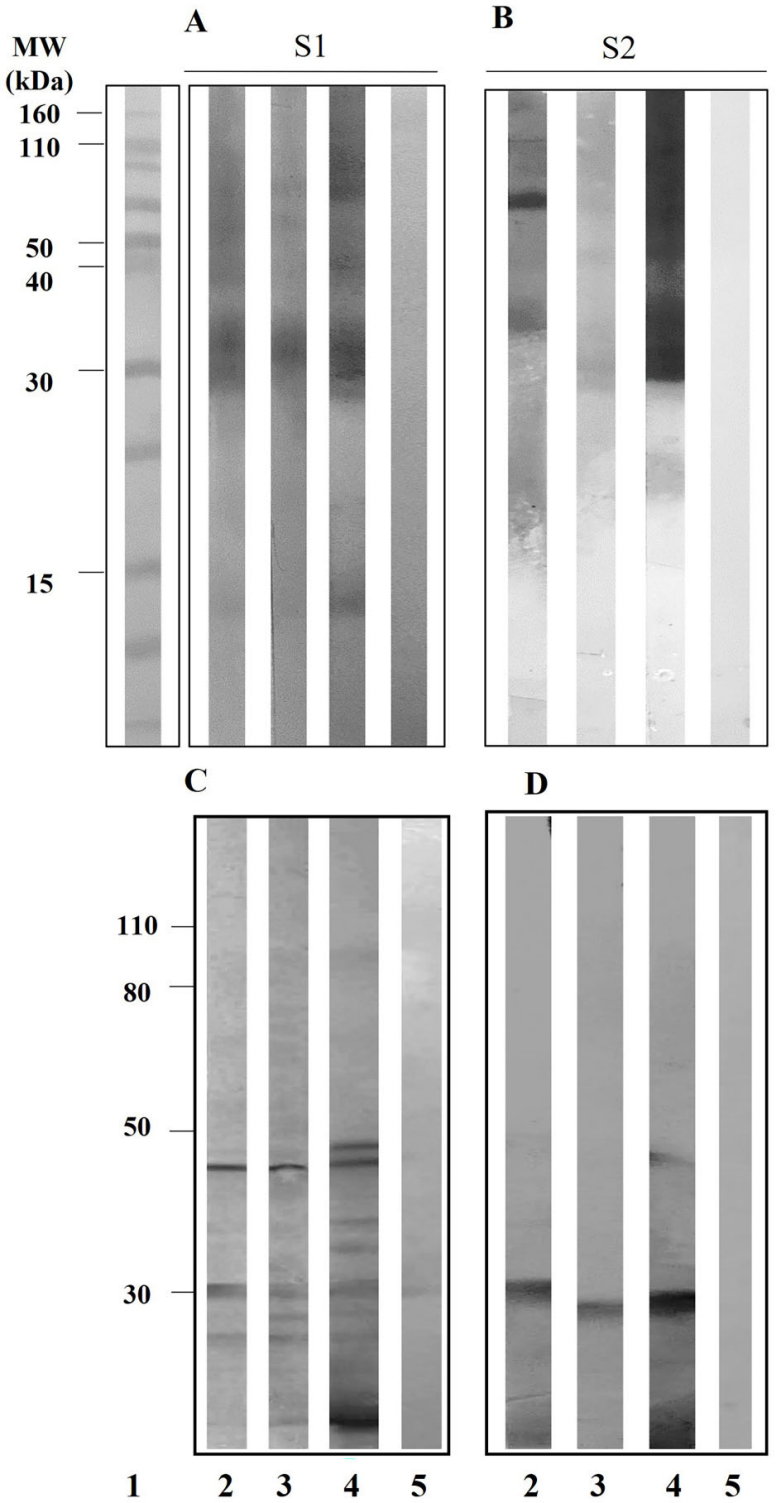
Representative immunoblotting of *Lolium multiflorum* total extract S1 and fraction S2 components recognized by IgE (**A** and **B**) and IgG4 (**C** and **D**) antibodies in three sera pools of atopic patients with pollinosis (lanes 2 to 4) and a sera pool of non-atopic subjects (lane 5). Protein standard molecular weight (MW) values are indicated in kDa (A, lane 1).

The reactivity analysis of *L. multiflorum* total extract S1 and fraction S2 components was evaluated in 35 serum samples of AT patients, which were divided into 7 pools (5 serum samples/pool), according to absorbance value obtained in ELISA for specific IgE antibody levels (maximum absorbance value: minimum absorbance value / number of pools). For negative control, the test was performed in 1 pool with 5 serum samples from NAT subjects. The representative images of immunoblotting for IgE antibody reactivity in AT patients (lanes 2-4) and NAT subjects (lane 5) are shown in Figure 4A (total extract S1) and Figure 4B (fraction S2).

**Figure 5:**
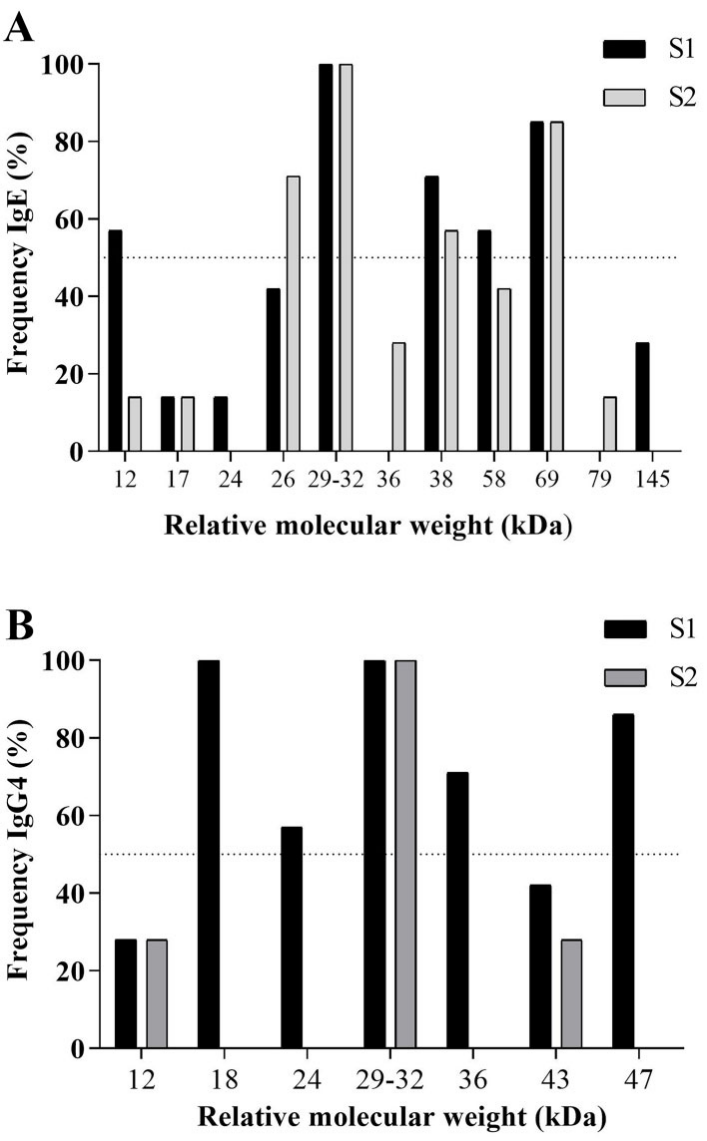
Frequency (%) of polypeptide bands of *Lolium multiflorum* total extract (S1) and carboxymethyl (CM)-binding fraction (S2) recognized by specific IgE (**A**) and IgG4 (**B**) antibodies in atopic patients with pollinosis, by immunoblotting. Dashed line indicates >50% of recognition.

The frequency of serum-specific IgE reactivity to the total extract S1 and fraction S2 are reported in Figure 5A. The 29-32 kDa band was the most recognized by serum pools of all AT patients when using either extract S1 or fraction S2 from *L. multiflorum*, followed by the 69 kDa band, recognized in 85.7% of the samples. Interestingly, bands of 12, 38, and 58 kDa were recognized by IgE antibodies predominantly in the total extract S1 whereas IgE reactivity to 26 kDa band was predominantly seen in the fraction S2. Other components with variable recognition were the bands ranging from 12 to 145 kDa as shown in Figure 5A. Serum samples of NAT subjects did not show any IgE reactivity to the extract S1 or fraction S2 components (Figure 4A and B, lane 5).

Also, the presence of specific IgG4 antibodies in AT patients was classified according to the absorbance values obtained in ELISA for specific IgG4 antibodies (maximum absorbance value - minimum absorbance value / number of pools). For negative control, the test was performed in 1 pool with 5 serum samples from NAT subjects (Figure 4C and D, lane 5). The representative images of immunoblotting for the reactivity of IgG4 antibodies to *L. multiflorum* total extract S1 and fraction S2 components in patients AT (lanes 2-4) are illustrated in Figure 4C (extract S1) and 4D (fraction S2).

The frequency-specific IgG4 reactivity to the total extract S1 and fraction S2 components are reported in Figure 5B. The 29-32 kDa band was the most recognized by serum pools of the AT group when using either extract S1 or fraction S2 from *L. multiflorum*. Four bands in the extract S1 were exclusively recognized by IgG4 antibodies, namely 18 kDa (100%), 47 kDa (85.7%), 36 kDa (71.4%), and 24 kDa (57.1%). Interestingly, no IgG4 reactivity was seen exclusively to components of fraction S2. Other components with variable recognition were the bands of 43 kDa (42.9 % in S1; 28.6% in S2) and 12 kDa (28.6% in S1; 28.6% in S2).

### LC-MS/MS characterization and properties of the main bands of fraction S2

**Figure 6:**
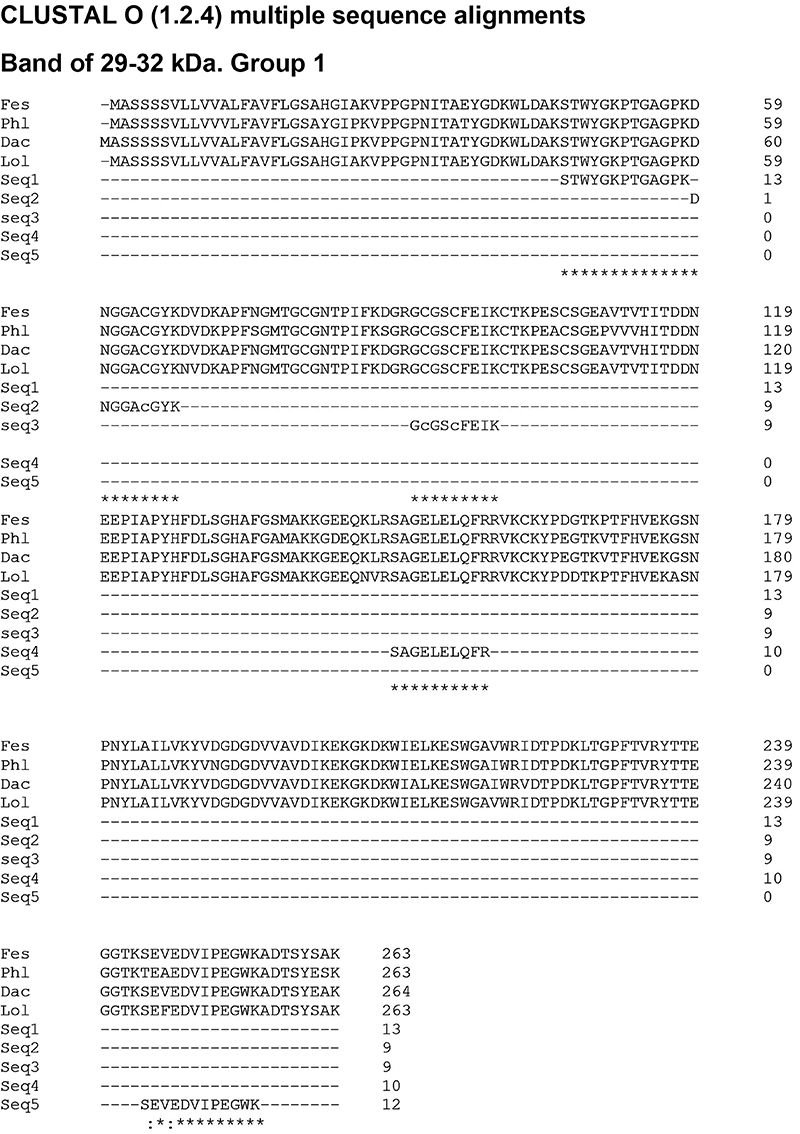
Sequence alignment of carboxymethyl (CM)-binding fractions derived from *Lolium multiflorum*. Samples compared with four FASTA sequences of several proteins obtained and aligned on the blastp (protein-protein BLAST) in the NCBI database. Fes: Fes p 1 (Sequence ID: CCD28289.1); Phl: Phl p 1 (Sequence ID: P43213.1); Dac: Dac g 1 (Sequence ID: AAP96760.1); Lol: Lol p 1 (Sequence ID: CAB63699.1). The equivalent amino acids in the analyzed proteins are marked with asterisks (*). A colon (:) indicates conservation between groups of strongly similar properties.

**Figure 7:**
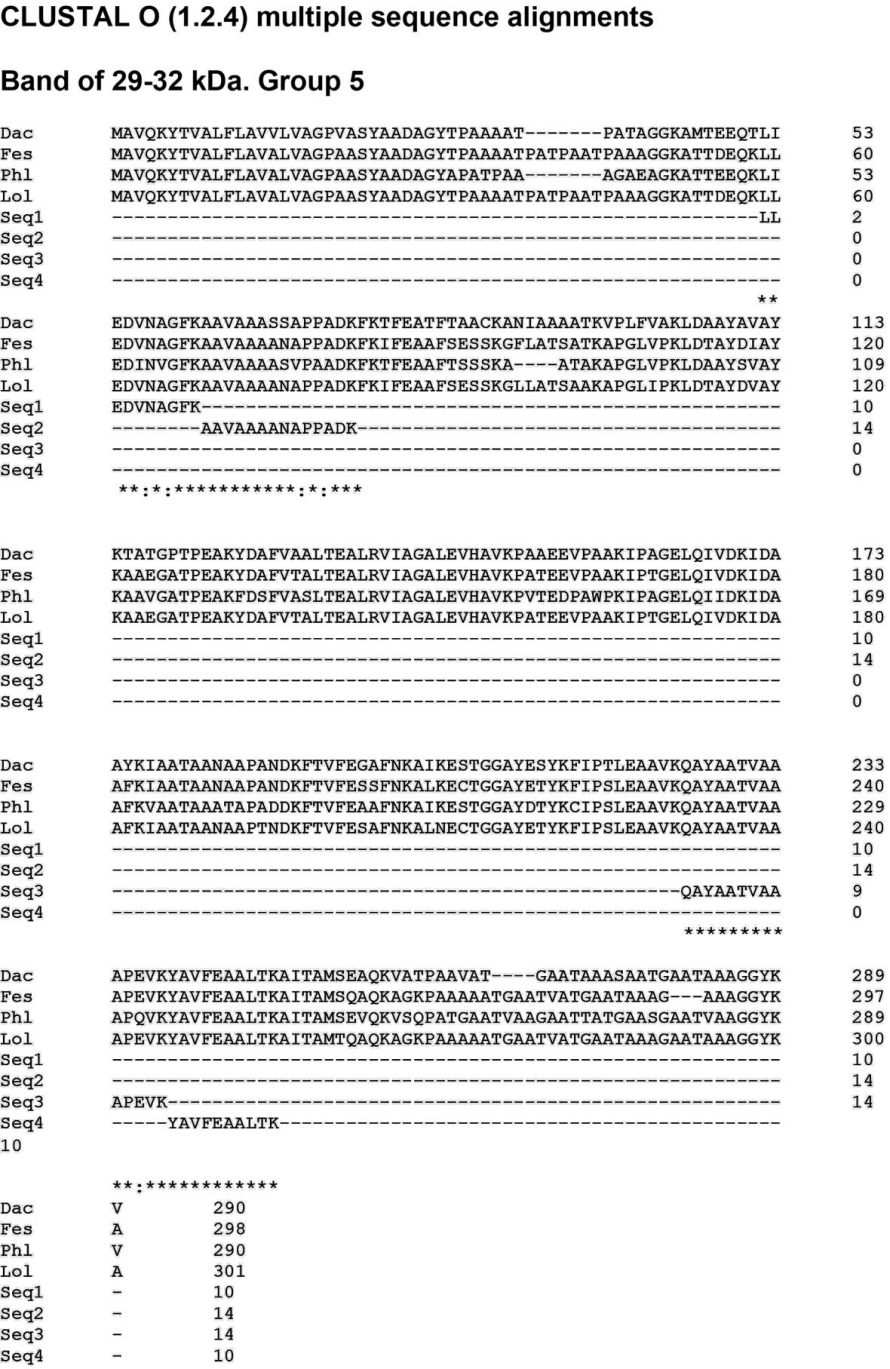
Sequence alignment of carboxymethyl (CM)-binding fractions derived from *Lolium multiflorum*. Samples compared with four FASTA sequences of several proteins obtained and aligned on the blastp (protein-protein BLAST) in the NCBI database. Fes: Fes p 5 (Sequence ID:CCD28288.1); Phl: Phl p 5 (Sequence ID:AAC25995.1); Dac: Dac g 5 (Sequence ID:AAK62278.1); Lol: Lol p 5 (Sequence ID: AAD20386.1).The equivalent amino acids are marked with asterisks (*). A colon (:) indicates conservation between groups of strongly similar properties.

**Figure 8:**
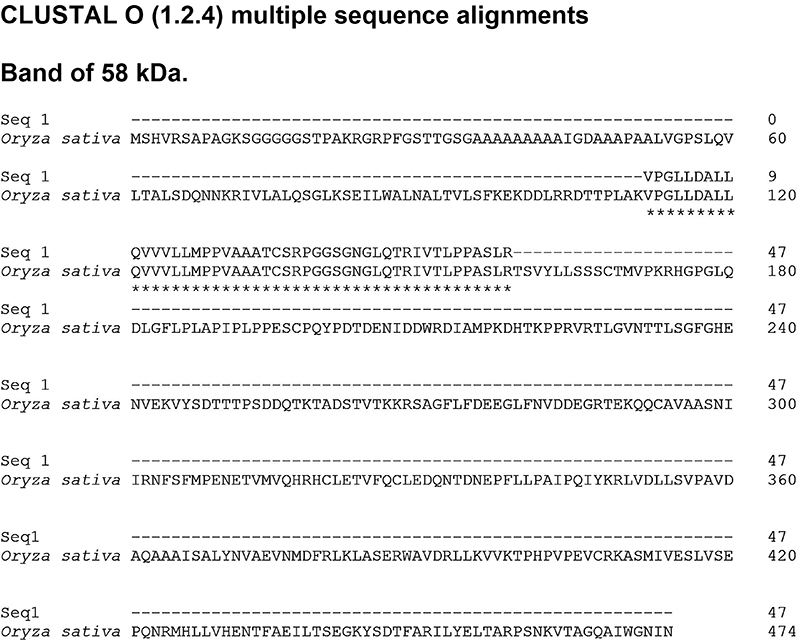
Sequence alignment of carboxymethyl (CM)-binding fractions derived from *Lolium multiflorum*. Samples compared with four FASTA sequences of several proteins obtained and aligned on the blastp (protein-protein BLAST) in the NCBI database. *Oryza sativa*: hypothetical protein *Oryza sativa* (Sequence ID: KAB8106316.1). The equivalent amino acids are marked with asterisks (*).

Searching the protein database at the National Center for Biotechnology Information (NCBI) with the sequences obtained from an analysis of the 29-32 kDa band, at least 3 UP sequences were revealed for 2 proteins like those found in other grasses. The first protein found presented 5 UP compatible with Lol p 1, among others β-expansins, and the second protein presented 4 UP sequences compatible with Lol p 5, and 3 other proteins from group 5 (Table 2), with specific differences in some amino acids of the sequences (Figures 6 and 7). On the other hand, only a long peptide from the 58-69 kDa band was identified in MS/MS analysis. This peptide displays 10% coverage with a hypothetical protein from *Oryza sativa* (Figure 8).

**Table 2:** Analysis by mass spectrometry of the main proteins recognized in the fraction S2 derived from *Lolium multiflorum* total extract.

Bands MW (kDa)	Protein	Organism	Similarity (%)/UP	Sequence ID(GenBank)
29-32	Lol p 1	*Lolium perenne*	5	CAB63699.1
-	Ph l p1	*Phleum pratense*	5	1N10_A
-	Dac g 1	*Dactylis glomerata*	5	AAP96760.1
-	Fes p 1	*Festuca pratense*	5	KAB8106316.1
29-32	Lol p 5	*Lolium perenne*	4	AAD20386.1
-	Ph lp 5	*Phleum pratense*	4	AAC25995
-	Dac g 5	*Dactylis glomerata*	4	AAK62278.1
-	Fes p 5	*Festuca pratense*	4	CCD28288.1
58-69	Hypothetical protein EE612_040618	*Oryza sativa*	(10%)	KAB8106316.1

MW: molecular weight; kDa: kilodaltons; UP: unique peptide.

## Discussion

*Lolium multiflorum* is one of the main allergen sources that elicit type 2 (T2) inflammatory response in subjects with IgE-mediated hypersensitivity. In the pathophysiological process involved in type I hypersensitivity, allergen proteases from *L. multiflorum* disrupt the epithelial barrier, thereafter captured by professional antigen presenting cells and presented to naive CD4^+^ T cells by co-interaction with T cell receptor (TCR) and MHC-II. Those cells will differentiate into Th2 cells by producing interleukin (IL)-5, IL-4, and IL-13 cytokines, which also play an important role in the recruitment of eosinophils and IgE production, which characterize the T2-high endotype (allergic eosinophilic pattern of the airway inflammatory diseases) (26). The IgE interaction with its receptor FcεRI will trigger allergic inflammatory immune response mediated by vasoactive amines, such as histamine, prostaglandin, leukotrienes, and platelet-activating factor, released by activated mast cells and basophils. Although the allergic response to plant allergens is well described, few studies are focused on *L. multiflorum*, which is associated with seasonal allergic rhinitis and asthma in several regions, including Brazil (10,11,27,28). Therefore, a better understanding of the specific immune response induced by this source of allergens that leads to severe pathological conditions becomes relevant, as is the development of diagnostic tools and allergen-specific immunotherapy approaches using biological products derived from *L. multiflorum*.

This study analyzed patients from southern Brazil who had been exposed to a variety of allergens during their lifetime, especially *Lolium multiflorum*, and reacted to pollen allergens from this grass. This was confirmed by SPT, with the average size of wheals larger in the AT group than the NAT group, as expected. Also, IgE- and IgG4-specific antibodies against *L. multiflorum* were detected using ELISA and immunoblotting techniques only in the AT group. These data are consistent with our previous studies (10,11). When evaluating the sensitization profile of the patients against *L. multiflorum*, serum IgE reactivity easily discriminated subjects from atopic and non-atopic groups, using either total extract S1 or fraction S2 derived from *L. multiflorum*, with no significant difference between them. By analyzing the electrophoretic profile of *L*. *multiflorum* total extract S1 and fraction S2, we observed that components with 12, 29-32, and 58 kDa were present in the highest intensity, particularly the band of 29-32 kDa in both fractions. The importance of these bands as major allergens was later confirmed by immunoblotting due to their high reactivity. Previous investigations have demonstrated that major components around 30 kDa are glycoproteins like β-expansins, which are related to papain-like proteases, corresponding to group 1 allergens (20,29-31).

Several studies have demonstrated that glycoproteins with molecular weights ranging from 25 to 37 kDa belonging to groups 1 and 5 allergens are the most important molecules involved in the induction of IgE antibodies to grass pollen, such as *L. perenne* and *P. pratensis* (11,29,32). Recently, Sterner and colleagues identified two isoforms of Cyn d 1, a β-expansin with about 29-30 kDa of *Cynodon dactylon* (Bermuda grass), by mass spectrometry as the major allergen in patients with allergic rhinitis in subtropical regions and responsible for cross-reactivity with other grasses in the same region (20,29,33). An additional Swedish study also revealed two β-expansins as the major sensitizing allergens in Phl p 1 and Cyn d 1 (33). These data indicate that β-expansins are the major group 1 grass pollen allergens. Our results, using mass spectrometry, revealed exactly the presence of group 1 proteins, β-expansins, and group 5 proteins, another important protein group. Therefore, we can sustain for the first time that ion exchange chromatography was able to enrich the major allergens derived from *L. multiflorum* and that the proteins likely responsible for cross-reactivity or other minor allergens did not link to the resin. As a result, these less relevant components were absent in fraction S2. This feature of the enriched fraction S2 may be considered more appropriate than the total extract S1 for allergy diagnosis since fraction S2 was able to keep sensitivity and specificity in ELISA comparable to the total extract S1. Therefore, we demonstrated the relevance of fraction S2 by inhibition ELISA. S2 revealed a high inhibition capacity similar to S1 and high-capacity binding to IgE and IgG4 specific for S1, which means that S2 kept with the main components of extract S1.

Furthermore, we can speculate that fraction S2 has a better indication than the *L. multiflorum* total extract for use in allergen-specific immunotherapy (AIT) since fraction S2 lacks many components that do not have clinical relevance (34,35). Although a previous study has shown that antibodies against *L. multiflorum* can highly cross-react with several other kinds of grass, including *P. pratense* and *C. dactylon* (11), several studies on AIT have demonstrated that it is always better to use a genuine allergen that contains clinical important allergenics and lacks components that are not relevant to atopic patients, because immunotherapy might induce new allergen sensitization. For this reason, it is more appropriate to use purified allergens, peptides, or fractions than crude allergenic extracts, particularly in AIT (36,37).

*L. multiflorum* total extract S1 and fraction S2 containing major allergens of 29-32 kDa, groups 1 and 5, showed strong specific IgG4 reactivity in both indirect ELISA and immunoblotting. A protective function against allergy has been greatly attributed to the generation of specific IgG4 subclass, which are antibodies that compete with IgE for binding to allergens and consequently decreasing mast cells and basophils activation by Fc_ε_ receptors, preventing histamine degranulation and allergic reactions (29,38,39). Currently, a total allergenic extract derived from mites, pets, and pollen is commonly used for both *in vivo* and *in vitro* diagnostic tests, as well as in AIT. In the last decade, studies have demonstrated that the use of purified peptides or proteins is also highly effective in AIT and promote an increase in IL-10-producing T-reg FOXP3+ cells, which are fundamental for the success of the treatment, because IL-10 is a crucial cytokine for inducing the production of specific IgG4 subclass in B cells, acting as a blocking antibody (39).

Altogether, we demonstrated that fraction S2 derived from total allergenic extract of *L. multiflorum* was able to bind to serum-specific IgE and IgG4 antibodies of atopic patients comparable to the total extract S1, and we observed that the 29-32 kDa band contained the main components of group 1 and group 5 allergens recognized by specific serum IgE and IgG4 antibodies of seasonal allergic patients.

The present study demonstrated the importance of identifying the major compounds involved in pollen sensitization of our patients living in southern Brazil. In addition, we have obtained a *L. multiflorum* allergenic fraction containing the most relevant allergens; however, we know that an isolated fraction cannot completely replace the total extract. Although fraction S2 contains the major allergens, the minor allergens might also be important for assessing allergies in patients and should be studied more in future research.

In conclusion, our findings indicated an alternative *L. multiflorum* allergen fraction (S2) that may be more appropriate than the total extract (S1) for diagnostic tests such as SPT or for the detection of specific IgE in *in vitro* assays, and might be a promising candidate for allergen-specific immunotherapy. For all these reasons, we hypothesize that fraction S2 may be a better candidate than the total extract S1 for allergen-specific immunotherapy in seasonal allergic patients sensitized to *L. multiflorum*. Finally, additional studies should be carried out to assess the effects of fraction S2 compared to the total extract in AIT, since fraction S2 can be derived from a relatively simple method for obtaining a fraction with the natural and genuine major allergens from *L. multiflorum* pollen.
